# Engagement With and Impact of an mHealth App for Childhood Obesity Prevention and Management: Protocol for a Mixed Methods Study

**DOI:** 10.2196/71551

**Published:** 2025-10-02

**Authors:** Madison Milne-Ives, Ananya Ananthakrishnan, Sophie R Homer, Jackie Andrade, Edward Meinert

**Affiliations:** 1 Translational and Clinical Research Institute, Faculty of Medical Sciences, Newcastle University, Newcastle-upon-Tyne, United Kingdom; 2 Centre for Health Technology, University of Plymouth, Plymouth, United Kingdom; 3 School of Psychology, University of Plymouth, Plymouth, United Kingdom; 4 Department of Primary Care and Public Health, School of Public Health, Imperial College London, The Faculty Building, Exhibition Rd, South Kensington, London, SW7 2AZ, United Kingdom, 44 191 208 6000

**Keywords:** telemedicine, mobile apps, mobile health, mHealth, pediatric obesity, healthy lifestyle, exercise, diet, behavior change, engagement

## Abstract

**Background:**

Childhood obesity is a serious global health concern that affects approximately 20% of children worldwide. Digital health behavior change interventions have the potential to improve behaviors that can contribute to childhood obesity, such as diet and physical activity, but often lack sufficient user engagement to achieve significant impact.

**Objective:**

The aim of this project is to develop evidence to better understand how users engage with digital interventions and how behavior change techniques can be leveraged to support engagement. Specifically, the study will examine the impact of a family-focused app for childhood obesity prevention on health behaviors, health outcomes, and communication between families and health care professionals (HCPs).

**Methods:**

A pretest-posttest, mixed methods evaluation will examine the impact of the NoObesity app on families’ physical activity and dietary behaviors and on HCPs’ self-efficacy at communicating with families about childhood obesity. Secondary outcomes will include well-being, usability, and users’ engagement with and perceptions of the intervention. An initial sample of 1000 families (children and young people of any weight and age under 18 years and their parents) and 180 HCPs will be recruited to participate in the study; a subset of these participants will be invited to take part in qualitative semistructured interviews. The study implementation and follow-up period will last for 6 months, with the outcomes measured at baseline and 3 and 6 months after baseline. Quantitative outcomes will be compared over time using repeated measures ANOVA, and qualitative data will be analyzed thematically and triangulated with app use data.

**Results:**

Ethics approval was granted by the Newcastle University Faculty of Medical Science Ethics Committee (2688/41816) on March 22, 2024. Recruitment has not yet started but will involve capturing informed consent (and assent from participants younger than 16 years).

**Conclusions:**

The project’s key contributions will be to generate evidence of the potential for a family-based digital intervention to support families’ health behavior change and HCPs’ confidence in their ability to support them and to improve our understanding of how particular behavior change techniques can be used to support engagement with the intervention and its target behavior. Findings will be disseminated through peer-reviewed journals and shared with the general public, with support from patient and public involvement representatives.

## Introduction

### Background

Childhood obesity is a growing, global public health concern [1,2]. Certain behaviors that contribute to obesity (eg, diet and exercise) are well-established factors in noncommunicable diseases such as cancer, heart disease, stroke, and type 2 diabetes [3], and obesity is strongly associated with increased likelihood of various mental and physical health conditions [3-5]. The ubiquity of cell phones and internet access in the general population has made digital technology a powerful, cost-effective means of supporting key health behavior interventions [6]. Mobile health apps have demonstrated the potential to support dietary and physical activity behavior change [7-10], but many do not have robust evidence of long-term positive impact [9,11-14]. A key limiting factor for impact is low engagement and adherence [15,16]. To achieve positive outcomes, there is a need to understand how digital tools for pediatric weight management can best support users’ engagement with interventions and their target health behaviors to achieve positive outcomes. This study will aim to assess the impact of such an intervention on health behavior change and explore how engagement can be best supported.

The worldwide prevalence of overweight and obesity is growing rapidly in children [17], particularly in countries such as the United States and the United Kingdom, where approximately 2 in 5 children are overweight or obese by the age of 11 years [18,19]. In the United Kingdom in 2015, approximately £6 billion (approximately US $9 billion) was being spent annually on obesity-related health care; by 2050, this is expected to increase to almost £10 billion (approximately US $15 billion) [20,21]. There are various determinants of childhood obesity—including factors that are difficult to modulate on an individual level, such as deprivation and genetics [22-24]—but behavioral factors such as diet and activity can influence weight and health outcomes [25-29]. Health care professional (HCP) involvement can help support family engagement with healthier behaviors, but weight can be a difficult and sensitive topic for them to discuss [30,31]. Barriers to effective communication include fear of upsetting or offending families and losing their trust, concerns about influencing eating disorders in children, stigma and negative attitudes about obesity, and time constraints [30-33]. A key factor affecting all of these barriers is a lack of sufficient education and training in the clinical skill of raising and supporting concerns about children’s weight [30-32].

As health care becomes increasingly digitized, digital tools such as mobile apps have the potential to provide widely accessible behavioral support; however, long-term benefit relies on sufficient engagement to achieve outcomes [13,34]. As studies have linked high levels of engagement with more positive health outcomes [35,36], improving engagement with digital behavior change interventions (DBCIs) could increase their potential impact [37,38]. Behavior change techniques (BCTs) [39] incorporated in DBCIs can help support engagement and impact [16,40], but evidence for associations between specific BCTs and engagement with DBCIs is limited [41], and there is a lack of clarity in the literature about which BCTs are most effective, in what contexts, and in what combinations [42].

This study will build on a recent examination of barriers and facilitators to parents’ engagement with a family-focused app for childhood obesity prevention and management (NoObesity) [43,44]. In line with previous digital health research, potential impact was limited by a lack of sufficient engagement to achieve the intervention aims (“effective engagement” [15]). Our previous evaluation identified factors influencing engagement and impact related to capability, motivation, and opportunity and provided theoretically and empirically grounded recommendations for addressing these barriers [43].

### Objectives

This study aims to develop evidence to better understand how users engage with DBCIs and how BCTs can be leveraged to support engagement, specifically in the context of obesity prevention and management, and to examine the impact of a family-focused app for childhood obesity prevention on health behaviors, health outcomes, and communication between families and HCPs.

## Methods

### Study Design 

**Figure 1.**
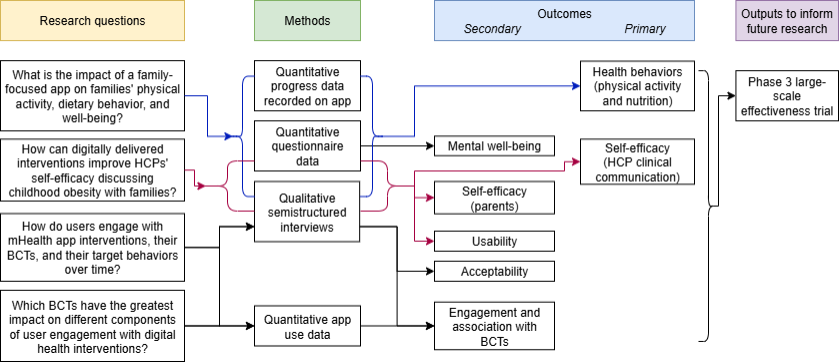
Study logic diagram. BCT: behavior change technique; HCP: health care professional; mHealth: mobile health.

The study will use an implementation science-centered approach to evaluate the impact of a family-focused app for childhood obesity prevention (NoObesity) on users’ engagement and behaviors and to gather feedback to enable further refinement of the intervention. A single-arm, pretest-posttest interventional study design will examine the impact of the app on families’ physical activity and dietary behaviors and HCPs’ self-efficacy at communicating with families about childhood obesity, how participants engage with the app, and how specific BCTs may be associated with engagement and impact. A single-arm study will allow the collection of a large dataset to explore engagement processes while also providing early evidence of potential health behavior change. The primary outcomes—family health behaviors and HCP self-efficacy—will be assessed with validated questionnaires (detailed in the *Outcomes* section). Mixed methods will be used to triangulate qualitative feedback from interviews with quantitative data to gather a more in-depth understanding of users’ engagement with the NoObesity app and their experiences using the app (Figure 1). The Standard Protocol Items: Recommendations for Interventional Trials (SPIRIT) checklist (Checklist 1) [45] was used to ensure the comprehensiveness of this protocol.

### Intervention

The NoObesity app was originally developed by Health Education England (now known as the South East School of Public Health, Workforce Training and Education Directorate, NHS England) in collaboration with the Universities of Bournemouth and Southampton to support childhood obesity management and prevention [46]. The aim of the intervention was to provide health behavioral support for families via goal setting, self-monitoring, and educational games and resources and to deliver training for HCPs on communicating with families about childhood obesity. Descriptions of the app’s features have been previously published [43]. The NoObesity app is being redesigned based on our initial evaluation [43] and the Behaviour Change Wheel [47]. The final version of the app that will be evaluated will have the same intended purpose as the original; its design and features will be detailed in the results paper.

### Patient and Public Involvement

Patient and public involvement groups, including parents and guardians, children, and HCPs, will be established to collaborate on the redesign of the app via recruitment on Voice UK and email invitations via clinical networks. Sessions will be held so that the patient and public involvement group members can guide the finalization of the app design and features, which will be incorporated in the version of the app evaluated in this study.

### Participants, Setting, and Eligibility

The target population of the study will include families—children and young people (≤18 years old) and their parents, regardless of their weight or BMI—and HCPs (Textbox 1). All eligible participants will be included. As the intervention is delivered digitally, participants can engage with it in various settings in their daily lives.

Textbox 1. **Eligibility criteria.**

**Inclusion criteria**

- All participants: willing and able to provide informed consent (aged >16 y) or assent to participate (aged <16 y), able to speak English, and own a mobile device capable of supporting the NoObesity app (iPhone; Apple Inc).
- Parents and guardians: parent or guardian of at least 1 child aged ≤18 years, of any weight or BMI.
- Children and young people: any age (up to and including children aged 18 y) and weight or BMI; no lower age limit will be set to enable parents to determine with their child whether they want to and feel capable of participating (eg, completing questionnaires and interviews).
- Health care professionals: working with families around weight, including but not limited to general practitioners, health visitors, school nurses, practice nurses, pediatricians, and pediatric nurses.

**Exclusion criteria**

- All participants: previous involvement in the development or testing of the NoObesity system, inability to provide self-consent (aged >16 y) or unwillingness to provide assent (aged <16 y), and a preexisting relationship with any member of the research team (eg, friends, family members, and colleagues).
- Children and young people: currently receiving specialist treatment for childhood obesity (tiers 3 or 4 obesity services).

### Outcomes

#### Primary Outcomes

To gather preliminary evidence of efficacy, the primary analysis for families will be health behavior change, specifically related to physical activity and dietary behavior (Table 1). These outcomes will be assessed using a combination of measures to enable triangulation and mitigate potential bias. This will include behavioral data captured via the app (self-report or sensor), repeated questionnaires, and qualitative feedback from semistructured interviews (SSIs). The Family Nutrition and Physical Activity Scale was selected because it examines the 2 main target behaviors (physical activity and healthy eating), has been validated for use with children aged ≤18 years, is easy to use, and is not too long [48,49].

**Table 1.** Primary and secondary outcomes and measures.

Outcomes		Participant	Outcome measure
Primary outcomes			
	Health behavior (physical activity and healthy eating)	Parents and CYP^a^	Self-reported goal progress in the app’s self-monitoring feature Linked phone or wearable sensors where captured (eg, step counts as measured by Apple Health or Fitbit) Qualitative feedback from SSIs^b^ Family Nutrition and Physical Activity [49]
	Self-efficacy	HCPs^c^	Self-Efficacy Questionnaire [50] Qualitative feedback from SSIs
Secondary outcomes			
	Engagement	All	App use data Uptake and dropout rates TWente Engagement with Ehealth Technologies Scale [51] Digital Behavior Change Intervention Engagement Scale [52] Qualitative feedback from SSIs
	Well-being	Parents and CYP	Warwick-Edinburgh Mental Wellbeing Scale [53]
	Self-efficacy	Parents	Qualitative feedback from SSIs
	Acceptability	All	Qualitative feedback from SSIs (structured using the Theoretical Framework of Acceptability) [54]
	Usability	All	mHealth^d^ App Usability Questionnaire [55] Qualitative feedback from SSIs

a CYP: children and young people.

b SSI: semistructured interview.

c HCP: health care professional.

d mHealth: mobile health.

For HCPs, the primary outcome will be the impact of the app on their perceived knowledge, skills, and confidence discussing obesity and weight-related topics with families. This will be examined using a self-efficacy questionnaire and explored in more depth in the qualitative SSIs. The Self-Efficacy Questionnaire was designed and validated to assess HCPs’ self-efficacy in the context of clinical communication skills training [50].

#### Secondary Outcomes

User engagement with NoObesity will be assessed using a combination of complementary measures [56], including system use data, questionnaires, and qualitative SSIs. Mixed methods approaches help capture the cognitive, behavioral, and affective components of engagement, providing a better understanding of users’ experience engaging with the intervention and the behavior change process [16,56-60]. Two recently developed measures of engagement with DBCIs are based on this multifaceted conceptualization and have similar psychometric properties: the Digital Behavior Change Intervention Engagement Scale [52] and the TWente Engagement with Ehealth Technologies Scale [51]. The scales are short (10 and 9 items, respectively), so both will be included as outcome measures to enable additional insights into user engagement [59]. We will also explore how engagement is associated with recruitment methods—whether participants found out about the study via their HCP or via social media advertisement—as previous research has found a positive relationship between clinical referral and engagement [61].

Other secondary outcomes will include usability (measured using the mHealth App Usability Questionnaire), self-efficacy and acceptability (explored in the qualitative SSIs), and well-being. Well-being will be included as a health outcome because of its positive correlation with improvements in physical activity and healthy eating [62]. The Warwick-Edinburgh Mental Wellbeing Scale [53] has been validated in many populations, including children and teenagers aged >11 years, so it is a good measure to capture parents’ perspectives and children’s and young people’s perspectives [63,64]. No weight-related measures will be captured; this is discussed further in the *Limitations* section.

### Sample Size

The target sample size was developed using a power calculation and expected dropout rates. On the basis of the literature, for the primary outcomes (Family Nutrition and Physical Activity Scale and Self-Efficacy Questionnaire), a sample of 199 families and 90 HCPs would achieve 80% power for an effect size of 0.2 and 0.3 (*α*=.05), respectively. High dropout is common across digital health research [65,66]; recent meta-analyses of mobile apps found pooled estimates of dropout rates between 40% and 50%. As our previous study had a high rate of attrition [43], we are aiming to enroll at least 1000 families, which would enable us to achieve the target sample size with 80% attrition. We will aim to recruit at least 180 HCPs to account for 50% attrition.

A subset of 20 to 30 participants will be selected to participate in SSIs using a stratified random sampling technique. This is within common estimates for qualitative sample sizes and in accordance with our capacity to conduct the study. Participants will be grouped based on demographic characteristics (eg, gender, ethnicity, and level of deprivation, which will be assessed by postcode [67]) and randomly selected from within these groups using a computer random number generator to achieve a diverse sample.

### Recruitment and Consenting Procedure

The study will be advertised using social media (Google Adwords and Meta Ads). After downloading the app from the Apple App Store, users will see an onboarding screen that includes a link to the study website. This will enable recruitment of people who came across the app in an app store without previously seeing recruitment materials.

Recruitment posters will be distributed to local general practitioner centers, children’s centers, schools, and other community organizations or networks. We will target centers and organizations in more disadvantaged areas, identified using the English Indices of Deprivation (2019) [68], to enhance sample diversity. This is a key target cohort to recruit, as there are strong links between obesity and deprivation [69]. The recruitment materials encourage potential HCPs to discuss the study with the families they work with and families to discuss the study with their HCPs to increase recruitment by word of mouth.

Recruitment materials will provide a brief overview of the study and link to the study website. Participant information sheets designed for various participant types—parents and guardians, children aged between 16 and 18 years, children aged between 10 and 15 years, children aged <10 years, and HCPs—will be accessible on the website to provide details about the study, including the potential benefits and risks; how data will be used, anonymized, and stored; and what participation will involve. No incentives will be provided for participation in the study, as we want to evaluate users’ engagement with the app without external incentives that would not be present in a real-world context.

Informed consent will be collected using the Qualtrics (Qualtrics International Inc) web-based software; if completed, the survey will continue to collect demographic data (eg, age, gender, ethnicity, and postcode as a proxy for the level of deprivation). Participants will also be asked how they found out about the study, as previous research has indicated that clinical referral is associated with engagement. For parents, the consent and demographics survey will include a question about the age or ages of their child or children. Depending on their response, a triggered email will be sent with the appropriate age-targeted participant information sheets and consent (for children aged between 16 and 18 y) or assent forms (for children aged <10 y and children aged between 10 and 15 y) so that they can discuss them as a family and email the research team with any questions they might have. Parents will complete informed consent on behalf of children <16 years, but 2 different simplified versions of an assent form will be provided for children aged <10 years and children aged between 10 and 15 years. All informed consent and assent forms will be delivered and completed through the Qualtrics platform.

### Data Collection

**Figure 2.**
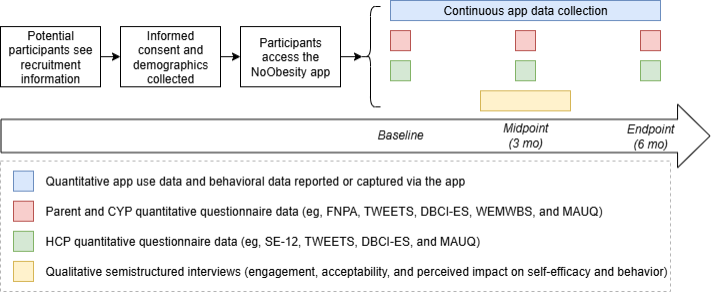
Participant flow diagram. CYP: children and young people; DBCI-ES: Digital Behavior Change Intervention Engagement Scale; FNPA: Family Nutrition and Physical Activity Scale; HCP: health care professional; MAUQ: mHealth App Usability Questionnaire; SE-12: Self-Efficacy Questionnaire; TWEETS: TWente Engagement with Ehealth Technologies Scale; WEMWBS: Warwick-Edinburgh Mental Wellbeing Scale.

Quantitative demographic data and outcome measures will be collected using a web-based survey platform (Qualtrics). All outcome measures will be captured at baseline, 3 months, and 6 months, except for the mHealth App Usability Questionnaire (captured only at 3 mo). App use data will be automatically stored through the app. SSIs will be conducted with a subset of users at approximately 3 months by the research team, who have no involvement in families’ care (Figure 2). A topic guide will provide a framework for the interviews (Multimedia Appendix 1 [16,54,57,60]). Interviews will be conducted using video or telephone conferencing software and recorded for transcription. If participants do not want the interview recorded, notes will be taken by hand and verified with the participant after the interview.

### Ethical Considerations

The ethics approval was received from the Newcastle University Faculty of Medical Science Ethics Committee (2688/41816) on March 22, 2024.

Written informed consent will be collected from participants via Qualtrics. The inclusion of children and young people as participants presents ethical considerations. As the app is designed for families, children and young people will be invited to participate alongside their parents or guardians to ensure that their perspectives are captured. Children aged <16 years will only be included if a parent or guardian provides informed consent and the child also provides assent; young people aged between 16 and 18 years will provide their own informed consent. Another consideration is the potential risk that topics arising in the interview may cause harm or distress (eg, triggering feelings of embarrassment or low self-esteem). To mitigate this risk, interview questions will primarily focus on participants’ experience with the app, which is not expected to be a sensitive subject, and questions related to behavior will specifically explore the app’s influence and not the types or extent of behaviors engaged in. Data privacy and confidentiality will be maintained as described in the following section.

### Data Management

Each participant will be given a unique identifier. Data will be analyzed using the unique IDs so that the overall dataset is pseudonymized. The transcription service receiving SSI recordings will only receive reference to the unique identifiers. The original audio recording will be destroyed after transcription. Records of consent will be kept for 10 years after the publication of final study results. All files containing participant data will be stored in the university’s secure Microsoft OneDrive.

The NoObesity system will store identifiable data in compliance with the UK Data Protection Act 2018. Organizations involved in this study will undertake a data protection impact assessment to ensure compliance with relevant data protection regulation, where needed. Participants will have the option to link external sensors (eg, Apple Health and Google Fitbit) to their app; only data included in the app will be anonymized and analyzed in line with other app use data.

### Data Analysis

Repeated measures ANOVA (or a nonparametric alternative, if initial checks identify that key assumptions are not met) will be used to compare differences between the means across the 3 time points (baseline, 3 mo, and 6 mo) for primary and secondary quantitative outcomes (health behavior, self-efficacy, and well-being). ANOVA is an appropriate method in this case, as it focuses on group-level trends over time, rather than subject-specific patterns. Missing data will be addressed through transparent justification of exclusions, and the context for missing data will be explored via qualitative analysis. This primary analysis will be conducted on the sample of participants who complete the outcome measures at each time point. Descriptive statistics will be used to evaluate the other quantitative outcomes (eg, usability scores and app use data). SSIs will be coded by 2 investigators using thematic analysis [70]. Triangulation of the quantitative questionnaire and system use data and qualitative SSI data will be conducted to validate the findings.

## Results

The study will be registered on ClinicalTrials.gov before recruitment begins. A series of co-design workshops were conducted with families and HCPs with lived experience to shape the redesign of the app from October to December 2024; these findings will be published separately. Development of this new version of the app is ongoing. An evaluation of the original version of the app is also published separately [43,44]. Recruitment and data collection dates will be finalized following completion of the app development process. As of September 9, 2025, no recruitment or data collection for the study has taken place.

## Discussion

### Limitations

Although the aim of the intervention is to support the prevention and management of childhood obesity, the study will focus on evaluating behavior change rather than weight-related outcomes. This is a limitation, as we will not capture evidence of clinical impact or determine if the app is associated with weight change. There were 2 main reasons for this decision. First, our aim is to evaluate the impact on engagement, HCP self-efficacy, and family behavior, in line with the app’s intended purpose. Although the overall aim of this behavior change is to mitigate childhood obesity, several participants in our previous evaluation reported feeling uncomfortable with weight measurements, especially for their children [43]. On the basis of this finding, we also expect that participants may be less likely to provide those data or participate if this measurement is required. Second, there is a logistical challenge in collecting high-quality data, as participants would have to self-report measurements, which introduces potential bias or inaccuracy if there are differences in scale calibration or performance of measurements. Likewise, the lack of a control group limits our ability to make causal inferences and introduces potential bias around external influences on the health behavior outcomes; however, a single-arm design will enable us to capture more data to understand engagement, which is a necessary prerequisite for behavioral and clinical impact. These limitations should be addressed in future, large-scale studies if the evaluation provides good evidence of the impact of the app on health behaviors.

Technical limitations of the app will affect generalizability. At present, patients without access to an iPhone will not be able to use the system and are therefore not eligible for inclusion in the study. If the study shows evidence of potential impact on health behaviors, we would aim to also create an Android (Google LLC) version of the app. In addition, as the app is currently only available in English, non–English-speaking patients will be excluded from the study, also limiting the representativeness of the sample.

There is also potential bias in the qualitative analysis from 2 sources: the influence of the interviewer on how participants respond (eg, social desirability bias) and the interpretation of participant responses. The first potential bias will be assessed by examining the balance of positive and negative feedback and by comparing the qualitative and quantitative data about NoObesity’s usability, acceptability, and impact. The second will be addressed by having multiple researchers conduct the thematic analysis to challenge individual perspectives and preconceived ideas.

### Conclusions

There is an urgent need to address the issue of childhood obesity. Family-based interventions have previously demonstrated positive impact on behavioral and health outcomes, particularly when involving HCP support [71], but this can be difficult to deliver on a large scale. This study will build on a previous evaluation of the NoObesity app by incorporating feedback and theory to improve the intervention and will assess its ability to positively impact families’ health behaviors and HCPs’ confidence in supporting them. We predict that engagement with the app will lead to improved physical activity, dietary behavior, and well-being for families and improved self-efficacy for HCPs. More broadly, we anticipate that this evaluation will provide useful insights into how users engage with DBCIs and how particular BCTs can support affective, cognitive, and behavioral engagement with the intervention and health behaviors, which will help inform future development of various DBCIs.

## Supplementary material

10.2196/71551 Multimedia Appendix 1 Sample topic interview guide.

10.2196/71551 Checklist 1 SPIRIT checklist.

## Abbreviations

BCT: behavior change technique
DBCI: digital behavior change intervention
HCP: health care professional
SPIRIT: Standard Protocol Items: Recommendations for Interventional Trials
SSI: semistructured interview
