# Supplementary material for: Engagement With and Impact of an mHealth App for Childhood Obesity Prevention and Management: Protocol for a Mixed Methods Study
Source: JMIR Res Protoc. 2025 Oct 2;14:e71551. doi: 10.2196/71551 (PMC12490775; doi:10.2196/71551)
Supplement: Multimedia Appendix 1 [file resprot-v14-e71551-s001.docx]

## Multimedia Appendix 1. Sample Topic Interview Guide

**Parents / guardians**

*Engagement with the app (including acceptability, usability)*

1. Overall, how did you feel about your experience using the NoObesity app? *(TFA - affective attitude [55])*
2. What motivated you to use the app? *(engagement - affective [16,58])*
   1. In what circumstances were you more likely to engage with the app? Ie. Why did you log on when you did? *(engagement - micro [61])*
3. How much did you use the app over the course of the study? *(engagement - micro, behavioural [61])*
   1. How did you use the app over the course of the study?
   2. What features of the app did you use the most / least?
4. How much effort did interacting with NoObesity take? *(TFA - burden [55])*
   1. How confident were you in your ability to use the app? *(TFA - self-efficacy [55])*
   2. What, if anything, made it difficult for you to use the NoObesity app?
   3. What, if any, technical issues did you have with the app? If you experienced any, how did that affect your experience?
   4. How well did you understand how NoObesity worked and how to use it? *(TFA - intervention coherence [55])*
5. How did using the app - or particular features of the app - make you feel? *(engagement - micro, affective [16,58,61])*
   1. What aspects of the app did you find most and least enjoyable? Why?
   2. What affected your enjoyment of the app features / content?
6. What did you think about the app’s features and content? *(engagement - micro, cognitive [16,58,61])*
   1. What aspects of the app did you find most interesting / caught your attention most? What aspects of the app did you find most boring? Why?
   2. What affected your interest in the app / content?
7. What concerns, if any, did you have about using the NoObesity app? *(TFA - ethicality [55])*
   1. How well or poorly did the NoObesity app align with your and your family’s values? *(TFA - ethicality [55])*

*Engagement with the behaviours*

1. Before you first used the app, how much confidence did you have in its ability to help you and your family achieve your goals and do healthier behaviours? *(TFA - perceived effectiveness [55])*
2. How much confidence did you have in NoObesity’s ability to support your health behaviour change? *(TFA - perceived effectiveness [55])*
   1. How well do you think NoObesity would work if used more extensively?
3. What helped motivate you to do your goals?
   1. What got in the way of your goals?
   2. What, if anything, about the intervention helped address those barriers?
4. How confident were you in your ability to achieve the goals you set? *(TFA - self-efficacy [55])*
5. What, if anything, did you have to give up to engage with the intervention? *(TFA - opportunity costs [55])*
   1. What, if anything, do you feel you gained from using the NoObesity app?
6. What, if any, changes to your thinking did you notice as a result of using NoObesity? *(engagement - macro [61])*
7. What impact did using NoObesity have on how you are going about changing your behaviour? *(engagement - macro [61])*
8. How do you think the NoObesity app did or did not impact your and your family’s behaviours?
   1. Why do you think this was the case?
9. What, if any, of the app features had the most impact on your engagement with your goals?
   1. Why do you think [feature] helped or hindered your engagement?
10. What would you suggest to make the app better?

**Healthcare professionals**

*Engagement with the app (including acceptability, usability)*

1. Overall, how did you feel about your experience using the NoObesity app? *(TFA - affective attitude [55])*
2. What motivated you to use the app? *(engagement - affective [16,58])*
   1. In what circumstances were you more likely to engage with the app? Ie. Why did you log on when you did? *(engagement - micro [61])*
3. How much did you use the app over the course of the study? *(engagement - micro, behavioural [61])*
   1. How did you use the app over the course of the study?
   2. What features of the app did you use the most / least?
4. How much effort did interacting with NoObesity take? *(TFA - burden [55])*
   1. How confident were you in your ability to use the app? *(TFA - self-efficacy [55])*
   2. What, if anything, made it difficult for you to use the NoObesity app?
   3. What, if any, technical issues did you have with the app? If you experienced any, how did that affect your experience?
   4. How well did you understand how NoObesity worked and how to use it? *(TFA - intervention coherence [55])*
5. How did using the app - or particular features of the app - make you feel? *(engagement - micro, affective [16,58,61])*
   1. What aspects of the app did you find most and least enjoyable? Why?
   2. What affected your enjoyment of the app features / content?
6. What did you think about the app’s features and content? *(engagement - micro, cognitive [16,58,61])*
   1. What aspects of the app did you find most interesting / caught your attention most? What aspects of the app did you find most boring? Why?
   2. What affected your interest in the app / content?
7. What concerns, if any, did you have about using the NoObesity app? *(TFA - ethicality [55])*
   1. How well or poorly did the NoObesity app align with your values? *(TFA - ethicality [55])*

*Self-efficacy*

1. Before you first used the app, how much confidence did you have in its ability to help you feel more confident and comfortable communicating with families about weight? *(TFA - perceived effectiveness [55])*
2. How well do you think the app’s training helped prepare you to talk with families about weight? *(TFA - perceived effectiveness [55])*
   1. How well do you think NoObesity would work if used more extensively?
3. How do you think the NoObesity app did or did not impact your belief in your ability to communicate effectively with families about weight?
   1. What features of the app did you find most useful?
4. How could the app better support your work with childhood obesity?

**Children and young people**

1. How did you use the app?
   1. By yourself? With a parent / guardian? With a sibling?
2. What did you think about the app?
   1. What was fun / made you feel good or happy? What wasn’t fun? Why?
   2. What did you find most interesting? What was most boring? Why?
3. Why did you want or not want to use the app?
   1. Did anything worry you about using the app? Can you tell me about it?
4. What, if anything, did you find difficult to do on the app?
5. Do you think that the app helped you do more healthy behaviours? How or why not?
   1. What do you think would have helped more?
6. How do you think we could make the app better?

**References**

16. Perski O, Blandford A, West R, Michie S. Conceptualising engagement with digital behaviour change interventions: a systematic review using principles from critical interpretive synthesis. Transl Behav Med. Jun 2017;7(2):254-267. [doi: 10.1007/s13142-016-0453-1] [Medline: 27966189]

55. Sekhon M, Cartwright M, Francis JJ. Acceptability of healthcare interventions: an overview of reviews and development of a theoretical framework. BMC Health Serv Res. Jan 26, 2017;17(1):88. [doi: 10.1186/s12913-017-2031-8] [Medline: 28126032]

58. Kelders SM, van Zyl LE, Ludden GDS. The concept and components of engagement in different domains applied to eHealth: a systematic scoping review. Front Psychol. 2020;11:926. [doi: 10.3389/fpsyg.2020.00926] [Medline: 32536888]

61. Short CE, DeSmet A, Woods C, et al. Measuring engagement in eHealth and mHealth behavior change interventions: viewpoint of methodologies. J Med Internet Res. Nov 16, 2018;20(11):e292. [doi: 10.2196/jmir.9397] [Medline: 30446482]
